# Vinculin and metavinculin exhibit distinct effects on focal adhesion properties, cell migration, and mechanotransduction

**DOI:** 10.1371/journal.pone.0221962

**Published:** 2019-09-04

**Authors:** Hyunna T. Lee, Lisa Sharek, E. Timothy O’Brien, Fabio L. Urbina, Stephanie L. Gupton, Richard Superfine, Keith Burridge, Sharon L. Campbell

**Affiliations:** 1 Department of Biochemistry and Biophysics and Lineberger Comprehensive Cancer Center, University of North Carolina at Chapel Hill, Chapel Hill, North Carolina, United States of America; 2 Department of Cell Biology and Physiology and Lineberger Comprehensive Cancer Center, University of North Carolina at Chapel Hill, Chapel Hill, North Carolina, United States of America; 3 Department of Physics and Astronomy, University of North Carolina at Chapel Hill, Chapel Hill, North Carolina, United States of America; 4 Department of Applied Physical Sciences, University of North Carolina at Chapel Hill, Chapel Hill, North Carolina, United States of America; Universitat Wien, AUSTRIA

## Abstract

Vinculin (Vcn) is a ubiquitously expressed cytoskeletal protein that links transmembrane receptors to actin filaments, and plays a key role in regulating cell adhesion, motility, and force transmission. Metavinculin (MVcn) is a Vcn splice isoform that contains an additional exon encoding a 68-residue insert within the actin binding tail domain. MVcn is selectively expressed at sub-stoichiometic amounts relative to Vcn in smooth and cardiac muscle cells. Mutations in the MVcn insert are linked to various cardiomyopathies. *In vitro* analysis has previously shown that while both proteins can engage filamentous (F)-actin, only Vcn can promote F-actin bundling. Moreover, we and others have shown that MVcn can negatively regulate Vcn-mediated F-actin bundling *in vitro*. To investigate functional differences between MVcn and Vcn, we stably expressed either Vcn or MVcn in *Vcn*-null mouse embryonic fibroblasts. While both MVcn and Vcn were observed at FAs, MVcn-expressing cells had larger but fewer focal adhesions per cell compared to Vcn-expressing cells. MVcn-expressing cells migrated faster and exhibited greater persistence compared to Vcn-expressing cells, even though Vcn-containing FAs assembled and disassembled faster. Magnetic tweezer measurements on Vcn-expressing cells show a typical cell stiffening phenotype in response to externally applied force; however, this was absent in *Vcn*-null and MVcn-expressing cells. Our findings that MVcn expression leads to larger but fewer FAs per cell, in conjunction with the inability of MVcn to bundle F-actin *in vitro* and rescue the cell stiffening response, are consistent with our previous findings of actin bundling deficient Vcn variants, suggesting that deficient actin-bundling may account for some of the differences between Vcn and MVcn.

## Introduction

Vinculin (Vcn) is an essential, ubiquitously expressed cytoskeletal protein that localizes to focal adhesions (FAs) and adherens junctions [1, 2]. It acts as a scaffold to link transmembrane proteins to actin filaments and plays a key role in cell adhesion, motility, and force transmission between cells and the cell-matrix interface. *Vcn* knockout mouse embryos do not survive past E10 and exhibit cardiac and neural tube developmental defects [3]. Additionally, *Vcn* null murine embryonic fibroblasts (MEFs) exhibit a more rounded morphology, increased motility [3, 4] and resistance to apoptosis and anoikis [5]. At the molecular level, Vcn is comprised of a large ~90 kD head domain, a flexible proline-rich linker, and a tail domain [6]. As part of its scaffold function, Vcn engages a number of cytoskeletal and adhesion proteins as well as phosphatidylinositol 4,5-bisphosphate (PIP_2_). The Vcn head interacts with talin at FAs, α-catenin at cell-cell junctions, and α-actinin at both cellular locations [7–10]. The proline-rich linker that connects Vcn head to Vcn tail can bind to a number of cytoskeletal proteins including VASP, vinexin, CAP/ponsin and the Arp2/3 complex [11–14]. Vcn tail directly binds to filamentous actin (F-actin) [15], PIP_2_ [16], paxillin [17, 18], and Raver1 [19]. Autoinhibitory interactions between the Vcn head and tail promote a closed inactive state, which obscures ligand-binding sites available to other interacting proteins [6]. Although mechanisms of activation are not fully understood, it is believed that engagement of talin or α-catenin to Vcn head in conjunction with binding of additional ligands such as actin [1, 8, 20], post-translational modifications [21], and/or mechanical tension [22–25], promote Vcn activation and scaffolding function by exposing multiple ligand binding sites.

Metavinculin (MVcn) is a larger splice isoform of Vcn that is selectively expressed in smooth and cardiac muscle cells and at low levels in platelets [26–28]. MVcn is expressed at sub-stoichiometric levels relative to Vcn (9–42%), and its expression correlates with the elevated contractile needs of these muscle cells [29, 30]. Complete knockout or heterozygous inactivation of the *Vcn* gene is associated with dilated cardiomyopathy in mice [31, 32], while reduced MVcn expression is also associated with dilated cardiomyopathy (DCM) and disorganized intercalated disc structures in humans [33]. Point mutations in MVcn have also been identified in patients with DCM and hypertrophic cardiomyopathy (HCM) [33–35]. While A934V and ΔL954 MVcn mutations are associated with DCM [34], an R975W mutation has been identified in patients with both DCM and HCM [35]. Both DCM and HCM are diseases of the myocardium that diminish blood flow within the heart due to reduced force transmission.

**Fig 1:**
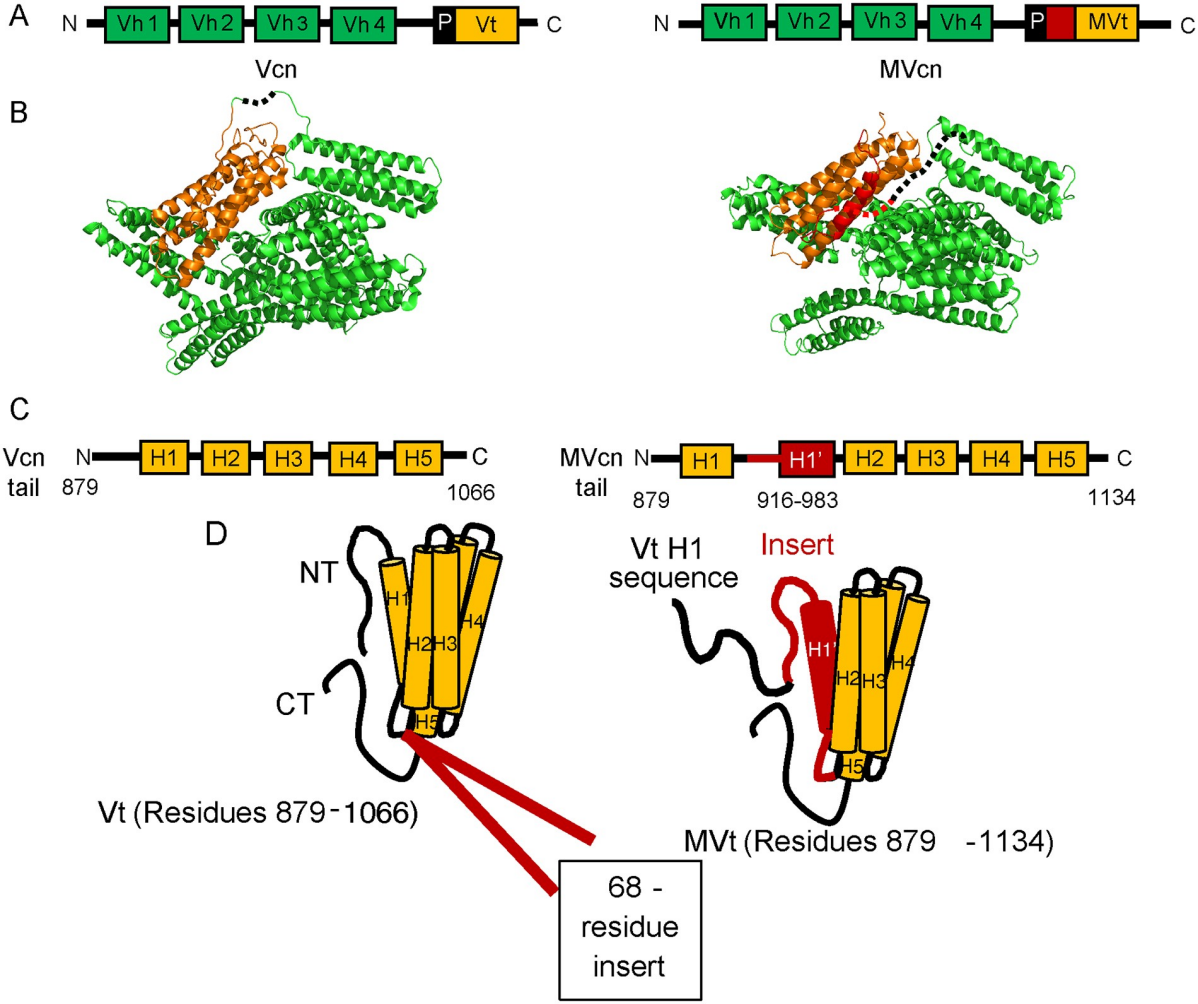
Sequence and structural differences between Vcn and MVcn. (A) Diagram comparing domain architecture of full-length Vcn and MVcn. (B) Crystal structures of Vcn (PDB: 1TR2) and MVcn [37]. Regions that lack electron density are represented by a dotted line. (C) Diagram comparing sequence differences in the Vcn and MVcn tail domain. MVcn tail contains an insert of 68 amino acid between residue 915 and 916 (H1’ in red, residues 916–983). (D) Structural schematic depicts sequence differences that lead to a helix replacement of H1 with H1’ (red) in MVcn tail (Vh: Vcn head domain; Vt: Vcn tail domain; MVt: MVcn tail domain (PDB:3MYI); H: helix; NT: N-terminus; CT: C-terminus; and P: proline-rich region).

MVcn and Vcn structurally share the same head domains [36, 37]; however, their tail domains differ. Vcn tail domain possesses an N-terminal strap followed by a 5-helix bundle and C-terminal hairpin [6], while the MVcn tail domain contains an additional exon that encodes a 68 amino acid insert [28]. While MVcn tail has a 5-helix bundle fold similar to Vcn tail, the sequence that makes up the helix 1 (H1) and strap of Vcn tail is displaced in the MVcn tail by homologous sequences, which we term H1’, contained within this insert [37] (Fig 1). Similar to Vcn tail domain, MVcn tail directly binds F-actin [37–39] but unlike Vcn tail domain, MVcn tail does not bundle filamentous actin into higher order structures *in vitro* [34, 38–40]. However, as MVcn and Vcn are co-expressed in muscle tissues [26, 28, 30], it is likely that they coordinately regulate actin filament organization. In fact, we and others have previously observed that the presence of MVcn tail at sub-stoichiometric ratios impairs Vcn tail-mediated F-actin bundling [39, 41], suggesting that MVcn tail may negatively regulate Vcn tail-mediated actin bundling.

While these differences in the ability of MVcn tail and Vcn tail to independently and coordinately reorganize actin networks have been observed *in vitro*, the field currently lacks a comparison of MVcn and Vcn in a cellular context. To investigate whether these two isoforms regulate distinct cellular functions, we stably expressed either MVcn or Vcn in a *Vcn*-null MEF background and compared FA properties, cell migration, and cell reinforcement to external force. Though we initially sought to use smooth muscle or cardiac cells as a system of comparison, the difficulty in maintaining and controlling for MVcn expression in cell culture prevented us from using those cells. In smooth and cardiac muscle cells, MVcn loses expression in cell culture unless the contractile environment is mimicked properly [30]. On the other hand, the *Vcn*-null MEF background allowed us to manipulate the expression levels of either Vcn or MVcn. We find that MVcn expression can fully rescue cell area and partially rescue FA number per cell. However, compared to Vcn-expressing cells, MVcn expression leads to larger individual FA area, faster cell migration, and decreased cell stiffening in response to external force. Our results suggest both overlapping and distinct cellular functions for MVcn and Vcn.

## Results

### MVcn-expressing cells have fewer but larger FAs compared to Vcn-expressing cells

**Fig 2:**
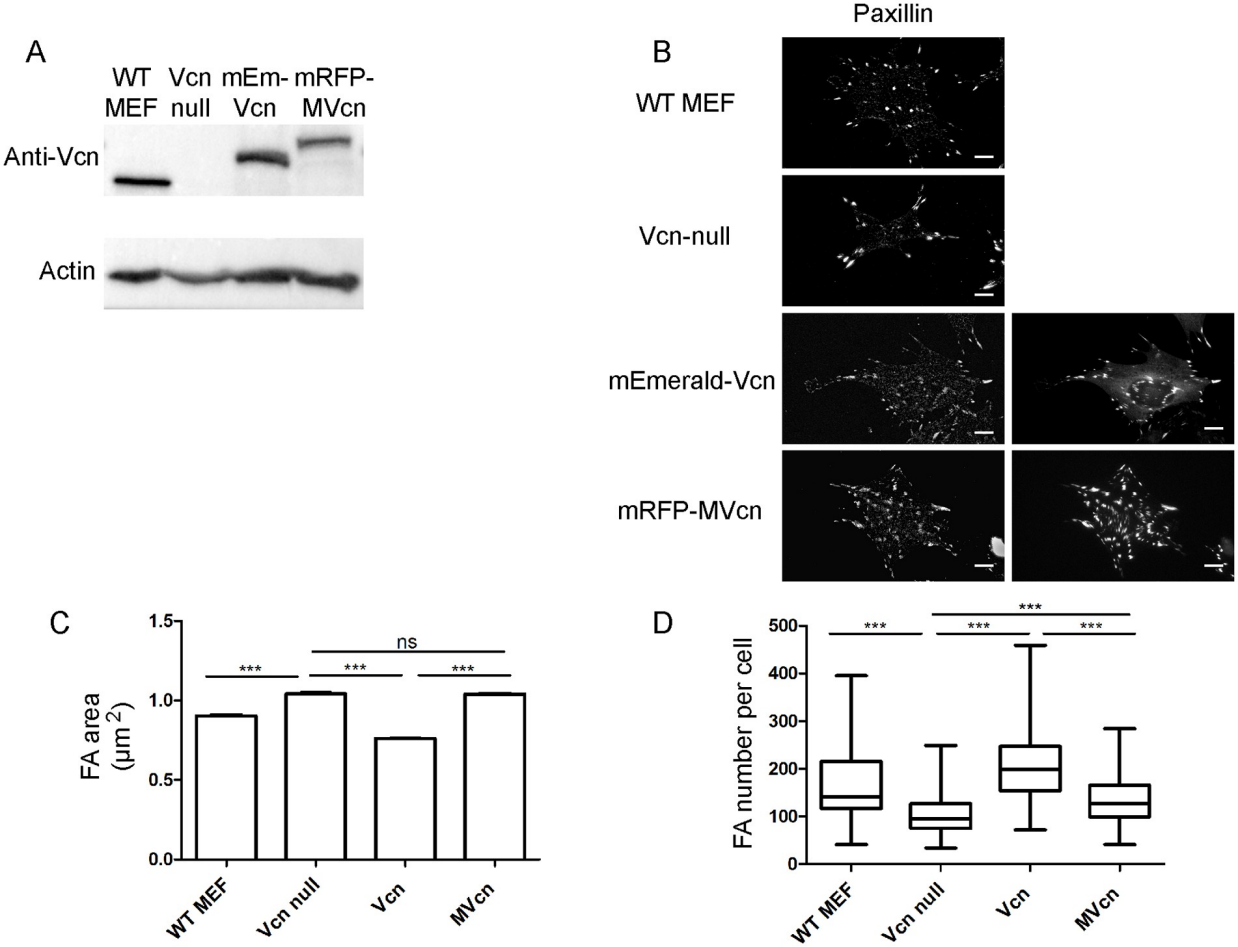
MVcn-expressing cells have larger but fewer FAs compared to Vcn-expressing cells. (A) Western blot shows the expression level of either Vcn or MVcn in Vcn null MEF background. Both expression levels are equivalent to endogenous Vcn expression in WT MEFs. (B) Fluorescent images of WT MEF, *Vcn*-null parent MEF cell line, exogenous Vcn-expressing, and MVcn-expressing *Vcn*-null MEFs stained for paxillin and showing expression of either fluorescently-tagged Vcn or MVcn. Scale bar = 10 μm. FA number (C) and overall FA area per cell (D) were quantified. Graphs represent data pooled from 4 independent experiments (n ≥ 100 cells; ***, p<0.001).

To establish a systematic comparison between Vcn and MVcn, we stably expressed either mEmerald-Vcn or mRFP-MVcn in Vcn-null mouse embryonic fibroblasts (MEFs). Because fibroblasts do not express endogenous MVcn, Vcn-null MEFs lack both endogenous Vcn and MVcn, providing a cell line that enables comparison of phenotypes associated with exogenously expressed Vcn or MVcn. To ensure similar expression levels of Vcn and MVcn, we used flow cytometry to select cells with Vcn or MVcn expression at levels equivalent to endogenous Vcn expressed in wildtype MEFs (Fig 2A; S1 and S6 Figs). We first confirmed that exogenously expressed Vcn and MVcn are both recruited to FA in the stable re-expressing cell lines (Fig 2B; S7 Fig). FA structures were identified by paxillin staining (Fig 2B). Interestingly, we observed that MVcn was recruited to FAs from the cytoplasmic pool approximately 2-fold more than Vcn.(S7 Fig).

As FAs are macromolecular structures that regulate cell adhesion, motility, and force response and transmission, we characterized FA properties of cells expressing either MVcn or Vcn, and compared them to the Vcn-null parent cell line. We employed immunofluorescence to quantify the mean FA area and the number of FA per cell using paxillin staining as a marker for FAs. The FAs of MVcn-expressing cells had larger areas but were fewer in number than the FAs of Vcn-expressing cells (Fig 2C and 2D). The mean FA area in MVcn-expressing cells was ~37% larger relative to that of Vcn-expressing cells (Fig 2C; Table 1). However, MVcn-expressing cells had 37% fewer FA per cell compared to Vcn-expressing cells (Fig 2D; Table 1). Overall, the FA properties of MVcn-expressing cells were more similar to the Vcn-null parent cells: FA size was not significantly different between MVcn-expressing cells and Vcn-null cells, although MVcn-expressing cells had slightly increased number of FAs per cell (30% increase) (Fig 2C and 2D; Table 1). Intriguingly, we have previously shown that a carboxyl-terminal deletion variant of Vcn that is defective in actin bundling shows similar FA properties to MVcn-expressing cells [42]. Furthermore, our observations that Vcn-expressing cells form smaller but more FAs per cell is consistent with previous findings that Vcn promotes FA formation [43].

**Table 1:** Quantified values of experimental results corresponding to Figs 2–6.

Experiment	Vcn null	Vcn	MVcn
FA area (μm^2^)	1.041±0.007	0.760±0.004	1.039±0.006
FA number per cell	103±3	212±6	134±4
Cell area (μm^2^)	818±27	1457±38	1481±41
Cell spreading rate (RTCA slope)	0.04	0.12	0.13
Cell Index (CI) at 2 hours	1.04±0.01	1.13±0.01	1.15±0.01
Velocity (μm/min)	0.84±0.03	0.29±0.01	0.52±0.02
Persistence	0.39±0.02	0.55±0.02	0.66±0.01
FA assembly rate (min^-1^)	N/A	0.139±0.004	0.110±0.004
FA disassembly rate (min^-1^)	N/A	0.125±0.003	0.093±0.003

### MVcn fully rescues cell area to the same extent as Vcn in Vcn-null MEFs

**Fig 3:**
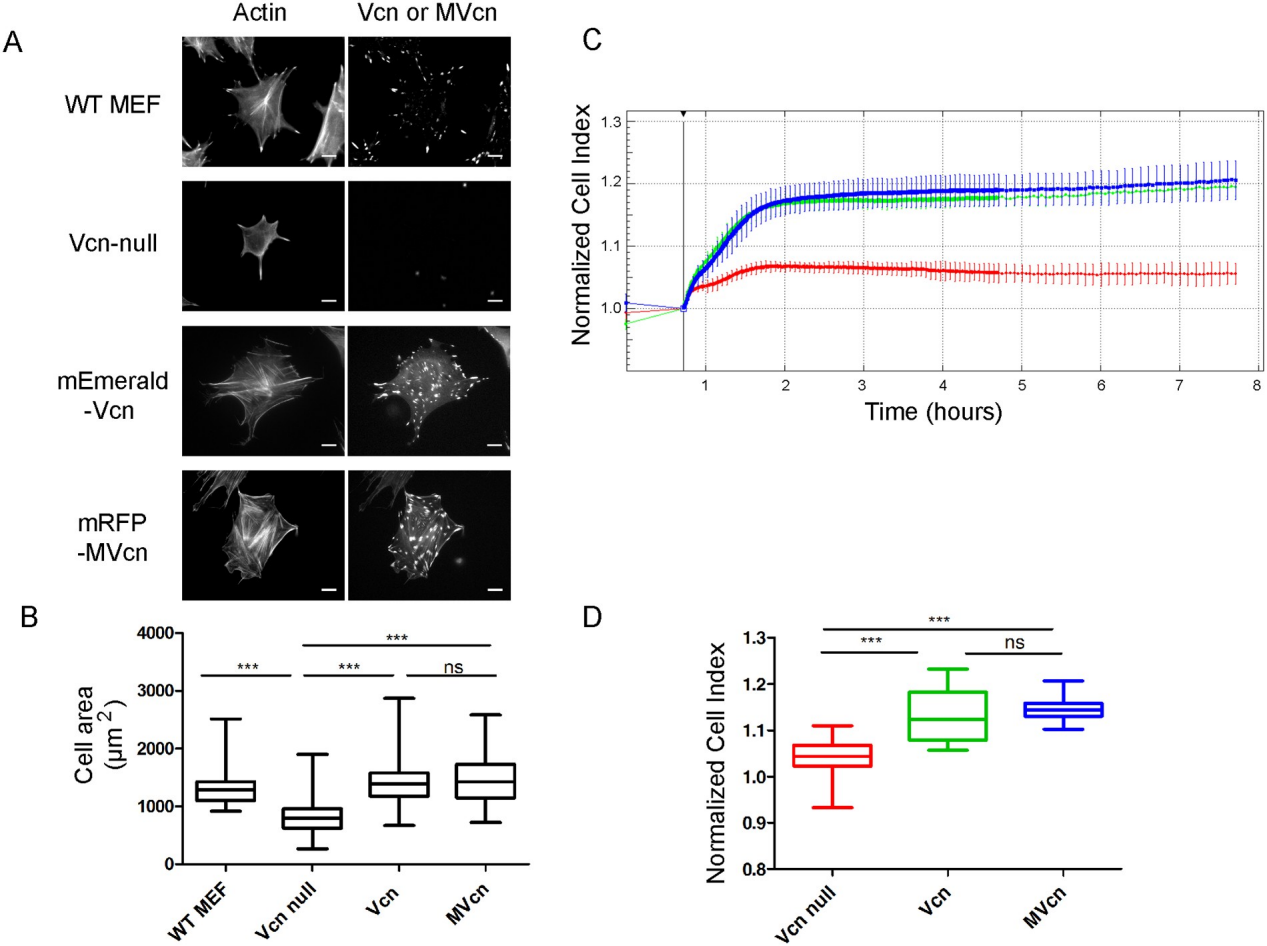
MVcn rescues decreased cell area in *Vcn*-null MEFs. (A) Stable fluorescently-tagged Vcn or MVcn expression in *Vcn*-null MEFs, co-stained with phalloidin, to allow cell area quantification. WT MEFs and *Vcn*-null MEFs stained with phalloidin and Vcn antibody are shown for comparison. (B) Cell area quantification. (n ≥ 150 cells per cell type, data pooled from 3 independent experiments; ***, p<0.001). (C) Representative real-time impedance traces from the RTCA xCELLigence system, measuring impedance every 15 s for the first 4 hours, and then every 3 min for the next 6 hours. Cell Index (CI) represents electrical impedance. First 8 hours are shown. CI is higher in cells expressing Vcn and MVcn compared to Vcn-null MEFs, indicating greater cell spreading. (D) Quantification of CI at 2 hours after seeding cells. Data pooled from 4 independent experiments (n ≥ 3500 cells per cell type; ***, p<0.001).

Given the differences in FA number and FA area observed between Vcn- and MVcn-expressing cells, we next investigated differences in cellular phenotype. *Vcn* deletion has previously been shown to significantly decrease cell size [4, 44], thus we examined whether re-expression of Vcn or MVcn could rescue cell area. *Vcn*-null, Vcn-expressing, and MVcn-expressing cells were stained with phalloidin to mark cell area (Fig 3A). Both Vcn- and MVcn-expressing cells had comparable cell areas that were significantly increased over *Vcn*-null cell area by 78% and 81%, respectively (Fig 3B; Table 1). Moreover, Vcn- and MVcn-expressing cells did not show significant differences in cell aspect ratios (S2 Fig). Upon finding that both Vcn and MVcn can rescue cell area in *Vcn*-null MEFs, we next quantified cell spreading on fibronectin (FN) using a real-time cell analyzer (RTCA) xCELLigence system. Compared to *Vcn*-null, cells expressing Vcn or MVcn had 10% higher Cell Index (CI), which represents electrical impedance, indicating increased cell area (Fig 3C and 3D). Additionally, the slopes of these traces provide real-time information on cell spreading rate. Based on this, Vcn-null cells have a slower spreading rate compared to both Vcn- and MVcn-expressing cells (~66%; Table 1). Thus, both Vcn and MVcn can fully rescue the decreased cell area of *Vcn*-null MEFs and show comparable cell spreading rates to Vcn-expressing cells.

### MVcn-expressing cells have faster migration velocity and higher persistence than Vcn-expressing cells

**Fig 4:**
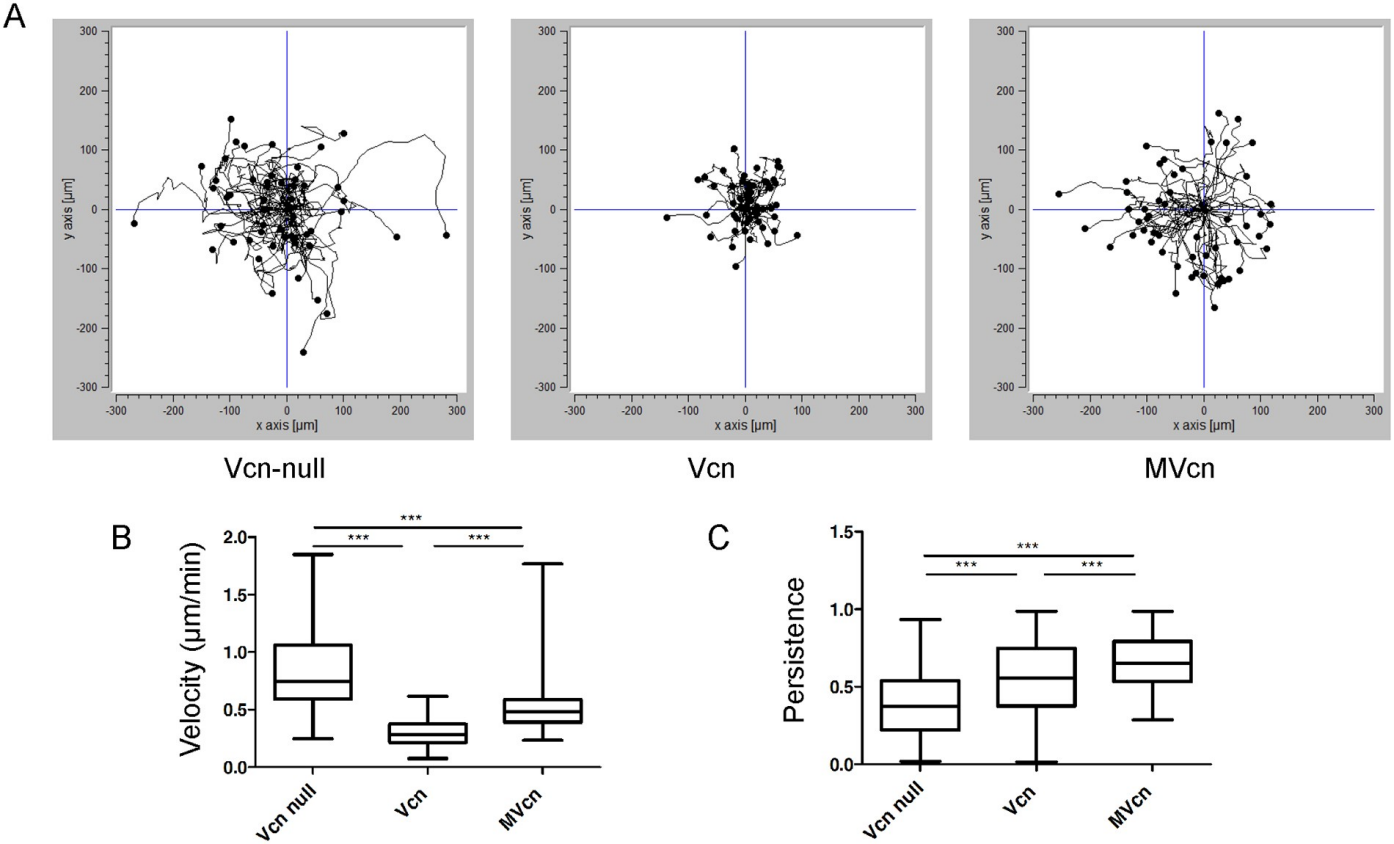
Random cell migration analysis shows enhanced migration velocity and higher persistence of migration for cells expressing MVcn compared to Vcn-expressing cells. (A) Representative cell tracks plotted for n>50 cells. (B) Quantification of random migration velocity. (C) Quantification of directional persistence. Data pooled from 3 independent experiments (n ≥ 140 cells; ***, p<0.001).

Vcn plays a key role in regulating cell motility as deletion of Vcn increases cell motility and random migration in 2D environments [3, 4, 45]. We assessed whether MVcn could restore a normal cell migration phenotype in the *Vcn*-null cell background. For *Vcn*-null, Vcn-, and MVcn-expressing cells, we monitored individual cell migration tracks every 15 minutes, manually tracking single cells and calculating both velocity and persistence via ImageJ [46]. Cell migration tracks show distinct migration paths for each cell type, with MVcn-expressing cells displaying longer migration tracks compared to the cells re-expressing Vcn (Fig 4A).

Cell migration velocity and cell persistence of the three cell types were also quantified. MVcn-expressing cells migrated faster than Vcn-expressing cells by ~78%, while *Vcn*-null cells migrated faster than either Vcn- or MVcn-expressing cells by ~184% and ~60%, respectively (Fig 4B; Table 1). Finally, we found that Vcn-expressing cells were more persistent than *Vcn*-null cells (~40%; Fig 4C; Table 1), consistent with observations from other groups [3, 45, 47]. Unexpectedly, MVcn-expressing cells were ~20% more persistent than Vcn-expressing cells and ~67% more persistent than *Vcn*-null cells (Fig 4C; Table 1).

### Vcn has faster assembly and disassembly rates at FA than MVcn

**Fig 5:**
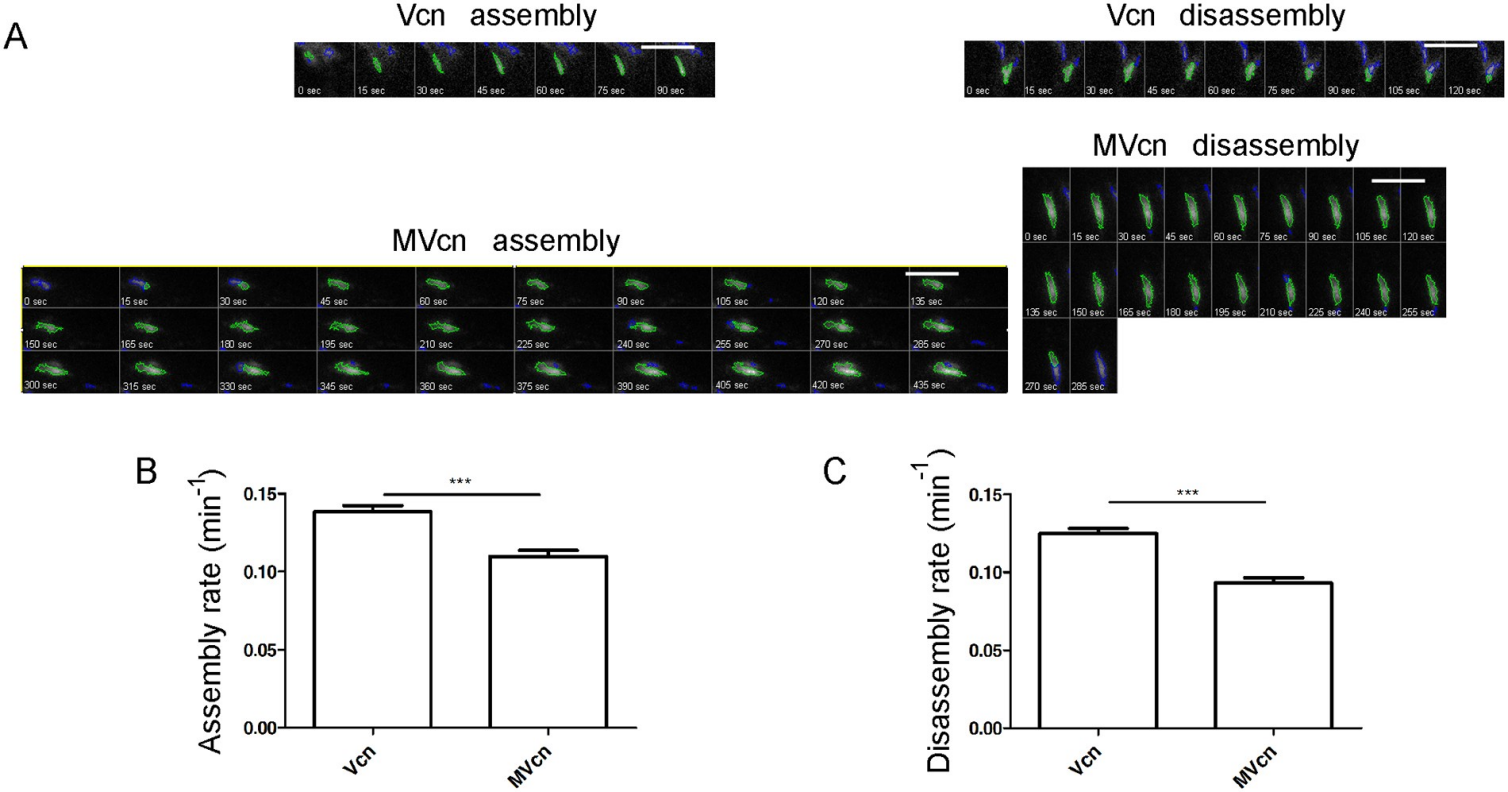
Focal adhesion assembly and disassembly rates are higher for Vcn-expressing cells compared to MVcn-expressing cells. (A) Representative time-lapse image sequences of Vcn-null MEFs stably expressing either mEmerald-Vcn or mRFP-MVcn migrating on 10 μg/ml FN. Images (shown in grayscale) are taken every 15 sec. Green outlines (generated by focal adhesion analysis program) show individual FA. Scale bar = 10 μm. Graph of average rate constants of FA assembly (B) and disassembly (C) from FAs in each cell type. Data pooled from 3 independent experiments (n ≥ 13 cells (and at least 400 adhesions); ***, p<0.001).

Focal adhesion assembly rate and turnover are highly dynamic events underlying cell migration [2]. Because the difference in average FA area and FA number per cell between Vcn- and MVcn-expressing cells suggested a potential difference in FA turnover, we examined FA dynamics of Vcn- or MVcn-expressing cells. Using total internal reflection fluorescence (TIRF) microscopy, we followed all of the adhesions within a single cell to quantify assembly and disassembly rates (Fig 5; Table 1). Vcn-containing FAs assembled at a faster rate (~26%) compared to MVcn-containing FAs (Fig 5B; Table 1). Likewise, Vcn-containing FAs showed a faster disassembly rate (~34%) compared to MVcn-containing FAs (Fig 5C; Table 1). The plots of fluorescence intensity with respect to time are also shown (S3 Fig). To ensure that the difference in fluorophores between Vcn and MVcn was not a contributing factor in measurements of FA assembly and disassembly rates, we tagged both proteins with the same mEmerald fluorophore and found the results to be consistent (S4 Fig; S1 Table). These results indicate that Vcn-containing FAs undergo faster FA turnover compared to MVcn-containing FAs, consistent with the previous findings that Vcn promotes FA turnover [43].

### MVcn-expressing cells lack a significant cell stiffening response to external force

**Fig 6:**
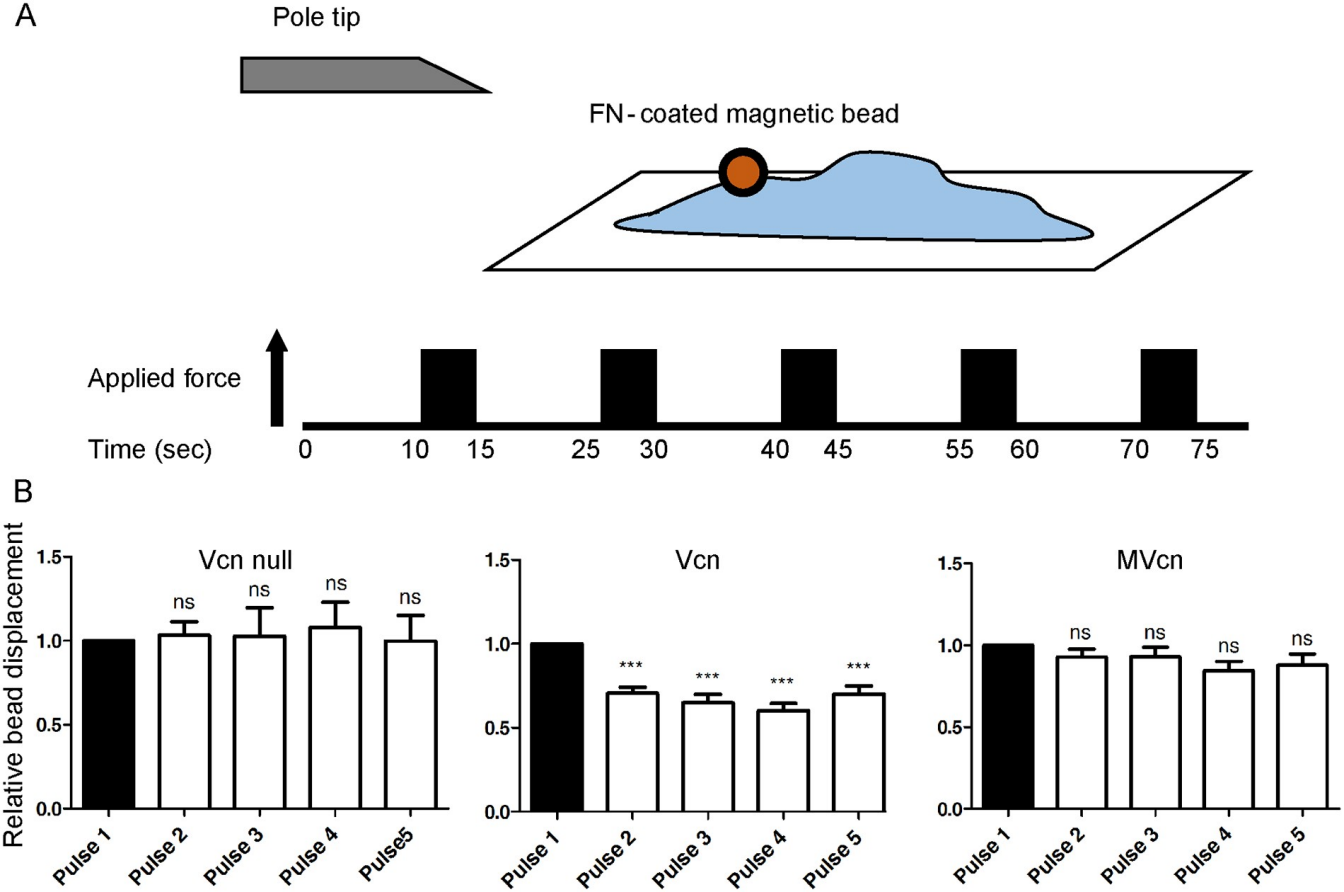
3D-Force microscopy (3DFM) shows reduced ability of MVcn-expressing cells to rescue cell stiffening response compared to Vcn-expressing cells. (A) 3D-force microscopy (3DFM) setup used to measure cell stiffening response. Constant force was applied for 5 sec intervals followed by 10 sec relaxation; this pattern was repeated for a total of 5 pulses. (B) Cell stiffening response is measured by quantifying the decrease in bead displacement after each subsequent pulse of magnetic force in *Vcn*-null, Vcn-, and MVcn-expressing cells. Cell stiffening response is lost in MVcn-expressing cells. Data pooled from 3 independent experiments (n ≥19 cells each cell type; ***, p<0.001).

Vcn is a mechanotransducing protein known to play an important role in force transmission by linking transmembrane receptors to the actin cytoskeleton [48–51], and knocking out Vcn leads to decreased traction force at FA [43]. Previous work demonstrated that Vcn bears force between the head and tail domains, and that the Vcn tail domain associates with actin filaments in cells, which has been shown to play an important role in cell traction force [25, 43, 52]. In addition, cells expressing Vcn were previously shown to respond to external force by displaying a cell stiffening response in 3D force microscopy (3DFM) (S5 and S9 Figs) while *Vcn*-null cells failed to show this response [25, 42, 53]. Given these findings, we investigated whether MVcn was likewise involved in force transmission. To study how Vcn- and MVcn-expressing cells respond to external force, we used 3DFM (Fig 6A) and assessed whether MVcn expression could restore the cell stiffening response in *Vcn*-null cells [25, 42, 53, 54]. To test this, we applied uniform pulses of force to cells via attached FN-coated magnetic beads. Decreases in the relative bead displacements between the first and subsequent pulses were quantified to determine the cell stiffening response. Here, we ensured that the actual magnetic bead displacements were similar between individual cells (S5 Fig). Vcn-expressing cells revealed a stiffening response, which was absent in the *Vcn*-null cells, as expected based on previous findings (Fig 6B) [42]. Specifically, *Vcn*-null cells failed to show decreased bead displacement after the first pulse while Vcn-expressing cells showed a 30% decrease in bead displacement between the first and second pulse of force (Fig 6B). In contrast, MVcn-expressing cells did not exhibit a significant bead displacement (8% decrease), indicating little or no stiffening response (Fig 6B).

## Discussion

Vcn and its splice isoform, MVcn, have been studied at both the tissue level and the molecular level [26, 29–32, 34, 37, 39, 41, 55]. However, a direct comparison of their behavior in cells is lacking. MVcn is co-expressed with Vcn at sub-stoichiometric levels in smooth and cardiac muscles, where the expression level correlates with the contractile needs of the cell [26, 27, 30]. The presence of MVcn in muscle cells is key, as mutations in the 68-residue insert lead to defects in the organization of intercalated discs and results in cardiomyopathy [34, 35]. These observations have led to the idea that MVcn coordinates with Vcn to support force transmission in cells [30, 34, 35]. At the molecular level, MVcn has been proposed to fine tune force transmission by negatively regulating Vcn-mediated actin bundling [41]. We and others have shown that *in vitro*, Vcn tail-mediated actin bundling is decreased as the MVcn tail concentration is increased [39, 41]. These findings suggest that MVcn may play a role in limiting, rather than strengthening, force transmission via Vcn tail-mediated actin bundling [39, 41]. MVcn tail has similar actin filament binding properties as Vcn tail, but does not dimerize and so does not bundle actin filaments [34, 37–41, 55]. These observations raise the following question. Is it possible that tail domain of MVcn competes with the Vcn tail domain in binding to actin filaments, providing a possible mechanism for modifying force transmission? However, before attempting to understand the role of MVcn in the context of Vcn, it is necessary to evaluate the properties of MVcn in cells. Herein, we investigated how stable expression of either Vcn or MVcn in a *Vcn*-null MEF background affects various cellular properties, including cell mechanotransduction.

Our findings indicate that some functions are shared between Vcn and MVcn at the cellular level, such as the recruitment of both proteins to FAs, and the ability of MVcn to partially rescue the number of FA per cell (Fig 2D) and fully rescue the cell spreading phenotype of Vcn-null cells (Fig 3). However, several distinct phenotypes including FA size, cell migration, FA dynamics, and cell reinforcement are observed (Figs 2D, 4, 5 and 6). These results suggest that similar phenotypes may be modulated by the shared head domain of the two proteins, while the distinct phenotypes may reflect their different tail domains.

The MVcn insert resides in the tail domain. Therefore, we were not surprised to find that both proteins are recruited to FAs, given that localization to FAs depends on the interaction of Vcn and MVcn head domains with talin [8, 28, 52, 56]. However, we did not anticipate to find MVcn enriched in FAs relative to Vcn when compared to their respective cytoplasmic levels. One possible explanation, consistent with findings from Chorev *et al*., is that the extra sequence in the MVcn tail favors the open “active” conformation [57]. This is also consistent with observations that the affinity between the head and tail domain of MVcn is weaker than that of Vcn, which likely facilitates talin engagement [55]. Moreover, it has been previously shown that constitutively active mutants of Vcn lead to larger, more stable FAs [52, 57, 58], a phenotype similar to what we see with cells expressing MVcn.

We and others have previously shown that while Vcn tail can organize actin filaments into parallel bundles, MVcn tail organizes actin filaments into a mesh-like network instead of bundles [34, 37–39]. The presence of the insert, including H1’, inhibits the ability of MVcn tail to bundle F-actin, as deletion of H1’ promotes actin filament bundling [37]. This difference in actin filament cross-linking between the two proteins likely plays a role in how cells regulate FAs and force transmission. Of note, we have previously shown that expressing an actin bundling-deficient Vcn mutant (VcnΔC5) in Vcn-null MEFs leads to defects in FA properties and decreased cell reinforcement in response to mechanical force [42]. As MVcn-expressing cells show similar defects in FA properties and force response, the inability of MVcn to bundle actin filaments might explain some of the differences observed between MVcn- and Vcn-expressing cells.

### MVcn expression alters FA properties

We found that FAs in MVcn-expressing cells have a larger area but are fewer in number per cell compared to Vcn-expressing cells (Fig 2). Similarly, we previously showed that VcnΔC5 expression in Vcn-null MEFs also results in larger mean FA area as well as fewer FAs per cell [42]. While a direct comparison cannot be made due to differences in both expression levels and cell type used for these studies, these FA trends strongly suggest the importance of actin bundling in FA regulation. Furthermore, MVcn-expressing cells are similar to Vcn-null cells in that they have larger FA size and fewer FA number per cell compared to Vcn-expressing cells. In fact, Vcn-null cells have the fewest FA number per cell out of all three cell types.

### MVcn expression affects mechanical response to force

In contrast to Vcn, expression of MVcn does not rescue the cell stiffening response in *Vcn*-null MEFs (Fig 6). 3DFM revealed that while Vcn-expressing cells showed almost immediate and significant decrease in bead displacement after the first pulse of force, MVcn-expressing cells did not show a significant decrease in bead displacement in response to successive pulses of tension (Fig 6). Similar to MVcn, when VcnΔC5, an actin bundling deficient mutant, was expressed in Vcn-null MEFs, cells were similarly defective in their stiffening response, additionally supporting that Vcn tail-mediated actin bundling is important for cell reinforcement [42]. It is possible that Vcn tail-mediated actin bundling is necessary for aligning actin in the regions of high tension. Recent studies using talin FRET sensors and cellular cryotomography demonstrated that regions of high talin tension had highly aligned linear actin filaments, while regions of low tension showed less well-aligned actin filaments in cells [59]. However, the ability of MVcn-expressing cells to maintain stress fiber structure suggests that the presence of other actin-crosslinking proteins, such as α-actinin and myosin, may contribute to the formation and maintenance of stress fibers within these cells [60, 61].

It is interesting to note that cells lacking Vcn show similar initial actual bead displacements (S5B and S8 Figs) compared to cells expressing Vcn. These findings are somewhat surprising, as they suggest that Vcn does not play a critical role in resisting the initial force applied to the fibronectin/integrin complex, in contrast to the widespread belief that Vcn functions as a critical mechanical connection between integrins (via talin) and actin filaments. While it is possible that the initial response is force dependent, (e.g. requires higher force), or that actin engagement by Vcn may require force, additional experiments will be needed to better understand these findings. Nevertheless, Vcn expressing cells do show decreased bead displacement in response to successive applications of force (Fig 6; S8 Fig), indicating the presence of Vcn is needed for this cell stiffening response, consistent with previous findings [25, 42, 43, 53].

### MVcn expression alters cell migration and FA dynamics

Does the inability of MVcn to bundle actin filaments account for most of the differences observed between Vcn- and MVcn-expressing cells? Our data showing differences in migration phenotypes suggest that other factors may be involved. While Vcn-null and MVcn-expressing cells migrated faster than Vcn-expressing cells, consistent with a negative regulatory function of MVcn tail, the migratory paths associated with MVcn-expressing cells displayed increased persistence relative to both Vcn-null and Vcn-expressing cells (Fig 3). MVcn in these cells also had slower assembly and disassembly rates in FAs compared to Vcn in Vcn-expressing cells (Fig 5). It has previously been shown that Vcn can facilitate FA formation and turnover [43], consistent with our findings that Vcn-expressing cells had smaller but more FAs per cell and faster FA assembly and disassembly rates, respectively. However, this is a curious result as increased FA turnover has been typically associated with faster cell migration, but the MVcn-expressing cells migrate faster than the Vcn-expressing cells. This could be partly due to differences in the ability of each isoform to engage particular binding partners. Moreover, the lack of Vcn-mediated actin bundling in MVcn-expressing cells may be compensated for by other actin-bundling proteins.

In conclusion, we find that MVcn can rescue some of Vcn’s functions in *Vcn*-null cells. Targeting FAs, it rescues cell area. Moreover, while MVcn partially rescues FA number, it is unable to fully rescue FA area. It also fails to restore the cell stiffening response to mechanical force in *Vcn*-null cells. Some of these differences may be due to the enhanced recruitment of MVcn to FAs or to the ability of MVcn and Vcn to engage distinct binding partners. However, the properties of MVcn-expressing cells are strikingly similar to those of cells expressing a Vcn mutant construct that can bind but not bundle actin filaments [42]. Consequently, we currently favor a model that attributes the different phenotype of the MVcn cells to be largely due to the inability of MVcn to bundle actin filaments. Future studies will continue to test this possibility.

## Experimental procedures

### Cell culture

WT MEFs and Vcn-null MEFs (from the same litter) were a gift from Dr. Brent Hoffman (Duke University), originally from Drs. Ben Fabry and Wolfgang Goldmann of the Erlangen Biophysics Group at the University of Erlangen-Nuremberg in Germany [45]. Human embryonic kidney (HEK) 293T cells were a gift from Dr. Channing Der at UNC. All cells were cultured in Dulbecco’s modified Eagle’s medium (DMEM; Invitrogen) supplemented with 10% fetal bovine serum (Sigma) and antibiotic-antimycotic solution (Sigma). They were grown in a 37°C incubator with 5% CO_2_.

### DNA constructs and generation of stable cell lines

mEmerald-Vinculin-23 was a gift from Michael Davidson (Addgene plasmid #54302; http://n2t.net/addgene:54302; RRID: Addgene_54302). mRFP-C1 was a gift from Robert Campbell & Michael Davidson & Roger Tsien [62] (Addgene plasmid #54764; http://n2t.net/addgene:54764; RRID: Addgene_54764). Human MVcn gene, a generous gift from Dr. Tina Izard, was cloned into RFP-C1 between SalI and ApaI restriction sites. mEmerald-tagged full-length human Vcn construct (1–1066) and mRFP-tagged full-length human MVcn construct (1–1134) were then subcloned into the pBabe-puro vector. Both fusion proteins were inserted using the restriction enzyme sites NgoMIV and SnaBI. Using the pBabe retroviral system to generate stable cell lines, we first generated retroviruses by transfecting HEK 293T cells with either the pBabe-puro mEmerald-Vcn or mRFP-MVcn constructs and the retrovirus packaging vector pCL-10A. pBabe-puro and pCL-10A vectors were gifts from Dr. Channing Der (UNC-Chapel hill). After 48 hours, the viruses were harvested and used to infect Vcn-null MEFs using 8 μg/mL polybrene. Vcn-null MEFs were infected for 24–48 hours and those expressing either mEmerald-Vcn or mRFP-MVcn proteins were selected with 7.5 μg/ml puromycin for a week. After the cells were kept under the selection pressure at 5 μg/ml puromycin for about 3 weeks, they were sorted for expression by flow cytometry. Expression levels of both mEmerald-Vcn and mRFP-MVcn were verified by Western blot analysis using anti-mouse vinculin antibody (Sigma), which recognizes both Vcn and MVcn, and HRP-conjugated anti-mouse IgG (Jackson). Actin bands were detected using a mouse anti-actin monoclonal antibody (Millipore).

### Flow cytometry

To select for established stable cells with consistent expression of mEmerald-Vcn or mRFP-MVcn, we sorted the established stable cells using a MoFlo XDP cell sorter from the UNC Flow Cytometry Core Facilty and chose cells with equivalent expression similar to physiological levels of Vcn. To ensure that any phenotypic differences between mEmerald-Vcn or mRFP-MVcn expressing cells were comparable, we tested the sorted cell populations for expression level via Western blot (Fig 2A) and chose cells with equivalent expression of mEmerald-Vcn and mRFP-MVcn equal to endogenous levels of Vcn in WT MEFs as a guide. The resulting stable cell lines allowed for physiological levels of expression of either construct in the same Vcn-null MEF background.

### Quantification of focal adhesion area and number per cell

Prior to FA analysis, cells were serum-starved in DMEM media supplemented with 0.5% delipidiated bovine serum albumin (BSA) and antibiotic-antimycotic solution. Cells were then trypsinized, resuspended in this same media, and rotated at 37°C for 2 hours before plating. Cells were seeded on glass coverslips coated with 50 μg/ml of FN and allowed to spread for 2 hours. Cells were then washed with phosphate-buffered saline (PBS), fixed in 3.7% formaldehyde for 15 min, and permeabilized in 0.2% TritonX-100 for 10 min in room temperature. Fixed cells were blocked with 5% BSA for 30 min at room temperature, and incubated with a mouse monoclonal anti-paxillin antibody (BD Transduction laboratory) for 1 hour. After washing with PBS, cells were stained with appropriate secondary antibody (either goat anti-mouse Alexa Fluor 488 (for mRFP-MVcn-expressing cells), or goat anti-mouse Alexa Fluor 568 (for mEmerald-Vcn-expressing cells) (Invitrogen) for an additional hour. Immunofluorescence images were then taken with a Zeiss Axiovert 200M microscope equipped with a Hamamatsu ORCA-ERAQ digital camera and 63x oil objective. A previously reported method [63] was adapted and used to identify and quantify the properties of paxillin-stained FA. This method applies a high pass filter to the images and a user-specified threshold to identify FA. Thresholded objects smaller than 10 pixels and larger than 200 pixels were excluded from analysis. FA number and size (area) was quantified for individual cells.

### Real-time Cell Analysis (RTCA) and cell spreading

The RTCA xCELLigence system (Acea Biosciences) uses electrical impedance to monitor the status of cells grown on micro-electrode coated plates [64]. With sparsely plated cells, changes in impedance reflect cell coverage of the substrate, i.e. cell attachment and spreading, reported as cell index (CI). Prior to seeding, cells were serum-starved in DMEM media supplemented with 0.5% delipidated BSA and antibiotic-antimycotic solution. Cells were then trypsinized, resuspended in this same media, and rotated at 37°C for 2 hours before being plated on 50 μg/ml FN-coated E-plate 16 wells. Cell Index was recorded at an interval of 15 sec for the first 4 hours, and subsequently at an interval of 3 min for the next 6 hours. Experiments were repeated 4 independent times with at least triplicates per sample. The slope of the RTCA trace was quantified from a trend line determined in Excel from data points between 0–2 hours post cell seeding.

To complement the RTCA data, cell area was also quantified by immunofluorescence. For the purposes of imaging, after plating for 2 hr, cells were fixed and incubated with either Alexa Fluor 488-phalloidin (for mRFP-MVcn cells) or Alexa Fluor 568- phalloidin (for mEmerald-Vcn cells) (Invitrogen) for 1 hour. Cell area was quantified using ImageJ [46]. Resulting data were represented as mean ± S.E.M.

### Random cell migration assay

Glass-bottomed culture dishes (MatTek Corp) were coated with 10 μg/ml FN at 37°C for 1 h. Cells were plated overnight before imaging. Cells were imaged at 37°C with 5% CO_2_ with a 10× objective on an Olympus VivaView FL microscope (Hooker Imaging Core at UNC) for 11 hours with 15 min intervals. Single cell tracking was manually performed in ImageJ [46] using the “Manual Tracking” plugin, in which cells are tracked based on the approximate centroid location over time. Only single cells were tracked and data was discarded if the cell experienced cell division, cell death, a collision event (with another cell or debris), or if it migrated out of the field of view. To obtain velocity and persistence values, raw tracking data were analyzed with the “Chemotaxis Tool” plugin (Ibidi) in ImageJ [46].

### Focal adhesion assembly and disassembly

TIRF images were collected on an Olympus IX81-ZDC2 inverted microscope equipped with a UAPON 100x/1.49NA DIC TIRF objective (Olympus), an automated XYZ stage (Prior) and an Andor iXon EM-CCD. Images were procured using the Metamorph acquisition software with 110 nm laser penetration depth. Time-lapse imaging was performed with a stage top incubator that maintained humidity, 37°C and 5% CO_2_ (Tokai Hit). Images were acquired every 15 sec for 30 minutes. Acquired images were further processed in ImageJ [46] to subtract background noise and to correct for photobleaching. A previously reported method [63] was used to identify and quantify the assembly and disassembly rates of the tracked focal adhesions within a single cell. This method applies a high pass filter to the images and a user-specified threshold to identify FA. Thresholded objects smaller than 10 pixels were excluded from analysis. All adhesions within a single cell were analyzed.

### Force microscopy

Three-dimensional force microscopy (3DFM) [65], a magnetic tweezer system, was used to apply consistent pulses of local 20–40 piconewton force to FN-coated magnetic beads, which were allowed to adhere to cells. Tosyl-activated magnetic dynabeads (2.8 um, Thermofisher) were washed with PBS and incubated overnight with FN at 37°C. After three washes with PBS, the beads were pipetted up and down vigorously to break up the aggregates and incubated with the cells for 20 minutes. Cells with 1 bead per cell were chosen for analysis. Constant force was applied for 5 sec intervals followed by 10 sec relaxation; this pattern was repeated for a total of 5 pulses. Upon the application of force, bead displacements were recorded with high-speed video camera (Jai Pulnix, San Jose, CA). Beads were tracked using Video Spot Tracker software [66]. The 3DFM system was calibrated prior to experiments using a fluid of known viscosity. We used 2.5 M sucrose as a fluid of known viscosity, which has a viscosity of 140 mPA-sec. Beads showing less than 10 nm of displacement (detection threshold for the 3DFM) were discarded for analysis. Custom Matlab scripts were used to calculate creep compliance, J_max_, also known as the deformity, which is defined as the average time-dependent deformation normalized by constant stress applied. $J_{max}=\frac{r_{max}}{F\times 6\pi \alpha }$, where r_max_ is the bead displacement due to the magnetic force F and *α* is the radius of the bead. Bead displacements were normalized to the bead displacement for Pulse 1 for each cell type and experiment and reported as mean ± S.E.M.

### Statistical analyses

Unpaired 2-tailed t-test was used for comparisons between two means. One-way ANOVA followed by Tukey post hoc was performed for multiple comparisons. All data were presented as mean ± S.E.M. unless otherwise noted. Statistical significance was set at *P<0.05; **P<0.01; ***P<0.001; P>0.05, not significant (n.s.).

#### Quantification of FA number per cell and FA area

One-way ANOVA followed by Tukey post hoc for FA number per cell was used to analyze significance. Unpaired 2-tailed student t-test was used for FA area analysis as one-way ANOVA with Tukey post hoc could not be performed because the number of groups is greater than 100.

#### Real-time Cell Analysis (RTCA) and cell spreading

Cell Index of all cell types were analyzed using one-way ANOVA followed by Tukey post hoc at 2 hours after seeding the cells.

## Supporting information

S1 Table Quantified values of FA assembly and disassembly rates from S1 Fig, for stably expressed mEmerald-Vcn and mEmerald-MVcn cells. (PDF)

S1 Methods (DOCX)

S1 Movie 3DFM force calibration in 2.5M sucrose. (AVI)

S1 Fig mEmerald-Vcn and mRFP-MVcn cells were sorted for expression level using flow activated cell sorting (FACS). For all panels, the top left figure represents population gated for cells of interest, top right figure represents population gated for doublet discrimination, and the bottom figure represents populations sorted based on the fluorescence intensity. (A) Sort data for Vcn-null MEF as non-fluorescent control for mEmerald-tag. (B) Sort data for Vcn-null MEF as non-fluorescent control for mRFP-tag. (C) Sort data for mEmerald-Vcn cell population. Population of high-expressing mEmerald fluorescence from gate R5 used for experiments. (D) Sort data for mRFP-MVcn cell population. Population of high-expressing mRFP fluorescence from gate R5 used for experiments.
(PDF)

S2 Fig Cell aspect ratio for all cell types. Data pooled from 3 independent experiments (n ≥ 90 cells); ***, p<0.001).
(PDF)

S3 Fig Average and representative assembly and disassembly curves at FA. All panels show fluorescence intensity plotted with respect to time as FAs either assembled or disassembled. For (A)-(D), all shaded areas indicate S.E.M. (A) Average assembly plot of mEmerald-Vcn at FAs for all cells. (B) Average disassembly plot of mEmerald-Vcn at FAs for all cells. (C) Average assembly plot of mRFP-MVcn at FAs for all cells. (D) Average disassembly plot of mRFP-MVcn at FAs for all cells. (E) Representative assembly plot of mEmerald-Vcn at FAs for a single cell. (F) Representative disassembly plot of mEmerald-Vcn at FAs for a single cell. (G) Representative assembly plot of mRFP-MVcn at FAs for a single cell. (H) Representative disassembly plot of mRFP-MVcn at FAs for a single cell. Data pooled from 3 independent experiments (n ≥ 13 cells (or at least 500 adhesions); *p<0.05; ***, p<0.001; not significant (n.s.)).
(PDF)

S4 Fig Focal adhesion assembly and disassembly rates display consistent results with the same tagged fluorophores. (A) Western blot shows equivalent expression level of either mEmerald-Vcn or mEmerald-MVcn in Vcn-null MEF background. (B) Representative time-lapse image sequences of Vcn-null MEFs stably expressing either mEmerald-Vcn or mEmerald-MVcn migrating on 10 μg/ml FN. Images are taken every 15 sec and show individual FA. Scale bar = 10 μm. Graph showing average rate constants of FA assembly (C) and disassembly (D) in each cell type. Data pooled from 3 independent experiments (n ≥ 13 cells (or at least 500 adhesions); ***, p<0.001).
(PDF)

S5 Fig 3DFM experimental set-up and controls. (A) Actual image of the experimental set-up. (B) Comparison of actual bead displacements between the first and second pulses for all cell types. Actual bead displacements between the first pulses of all cell types are similar. (C) Graph showing the relationship between the magnetic force experienced by the bead and the distance between the magnetic pole tip and the bead. Data pooled from 3 independent experiments (n ≥19 cells each cell type; not significant, n.s.).
(PDF)

S6 Fig Quantification for western blot in Fig 2A. (PDF)

S7 Fig Quantification for FA localization of mEmerald-Vcn and mRFP-MVcn. (PDF)

S8 Fig Paired data set for each cell type in S5B Fig between actual bead displacements of first and second pulse. (A) Data from S5B Fig showing statistical significance between the actual bead displacements of first and second pulses for each cell type. Paired data set for actual bead displacements between first and second pulse of (B) Vcn null MEFs, (C) Vcn-expressing MEFs, and (D) MVcn-expressing MEFs are shown. Same data from (A), S5B Fig, and Fig 6 used for the analysis of (B)-(D).
(PDF)

S9 Fig Images of 3DFM set-up showing the microscope and the magnet. (A) Image of 3DFM microscope with cells. (B) Image of experimental set-up after the magnet has been placed.
(PDF)
