# Supplementary material for: Vinculin and metavinculin exhibit distinct effects on focal adhesion properties, cell migration, and mechanotransduction
Source: PLoS One. 2019 Sep 4;14(9):e0221962. doi: 10.1371/journal.pone.0221962 (PMC6726196; doi:10.1371/journal.pone.0221962)
Supplement: S1 Table — (PDF) [file pone.0221962.s001.pdf]

S1 Table. Quantified values of FA assembly and disassembly rates of mEmerald-Vcn and mEmerald-MVcn stably expressing cells.

Experiment	mEmerald-Vcn	mEmerald-MVcn
FA assembly rate (min^{-1})	0.137±0.003	0.099±0.004
FA disassembly rate (min^{-1})	0.128±0.002	0.095±0.003
