# Supplementary material for: Vinculin and metavinculin exhibit distinct effects on focal adhesion properties, cell migration, and mechanotransduction
Source: PLoS One. 2019 Sep 4;14(9):e0221962. doi: 10.1371/journal.pone.0221962 (PMC6726196; doi:10.1371/journal.pone.0221962)
Supplement: S1 Methods — (DOCX) [file pone.0221962.s002.docx]

**Supporting Methods**

***Generation of average and representative assembly and disassembly rates at FA.***

Time series graphs of each cell were created by first temporally aligning the start of each focal adhesion assembly or disassembly phase. The average fluorescence of each focal adhesion was then plotted +/- 95% confidence interval generated through non-parametric bootstrap samples (n = 1000) for obtaining confidence limits for the population mean without assuming normality.

For combining all cell focal adhesions, each focal adhesion assembly or disassembly phase was temporally aligned and normalized to the starting fluorescent values. The normalized average values of each focal adhesion were then plotted as above,  +/- 95% confidence interval generated through non-parametric bootstrap samples (n = 1000) for obtaining confidence limits for the population mean without assuming normality. The following are references for the programs and the packages used.

*References:*

Hadley Wickham (2007). Reshaping Data with the reshape Package. Journal of Statistical Software, 21(12), 1-20. URL <http://www.jstatsoft.org/v21/i12/>.

Wickham H (2016). ggplot2: Elegant Graphics for Data Analysis. Springer-Verlag New York. ISBN 978-3-319-24277-4,

R Core Team (2017). R: A language and environment for statistical computing. R Foundation for Statistical
Computing, Vienna, Austria. URL [http://www.R-project.org/](https://www.r-project.org/)

***Calibration of Magnetic Force for Force Microscopy***

Once we know the pole position, we determined what forces are produced at different distances from that point at a given voltage. Voltages were then applied in a series of 1s on 1s off pulses to a suspension of 2.8 um Dynabeads in a 2.5 M sucrose solution. Using FIJI plugin MTrackJ and the calibration values for the camera (0.40 um/pixel and 0.034 sec between frames), the movement of a bead was tracked as it was drawn in toward the pole (S1 Movie; Panel C from S5 Fig). Different colors represent the bead movement per pulse of magnetic force application. The graph in panel C of S5 Fig shows the relationship between force and distance directly. Note that the relationship is not linear, but increases rapidly. Some estimates are that the force decreases as the 5^th^ power of distance. Thus at 90 µm from the tip (average distance between bead and the magnetic pole tip), about 20-40 pN of force is produced.

***Determination of Magnetic Force for Force Microscopy***

Our approach to calculating the forces produced by our magnetics system is to record at high frame rate the movement of representative beads through a non-elastic (“Newtonian”) fluid of known viscosity. In these experimental conditions, the bead experiences low Reynolds number (*Re*) flow profile, where *Re <* 10^−4^. Here, the “ma” term from F=ma is negligible, leaving the force to scale approximately linearly with viscous drag or “mv”. This is captured by the Stokes/Einstein equation, $F=3\pi d\eta v$, where *η* is the viscosity of the fluid and *v* is the velocity of the sphere. Using the 2.8 um beads and 2.5 M sucrose (viscosity: 140 mPA-sec), we find that: F = 3*(3.1415)(1.4 m^-6^) (0.14 N-s/m^2^)(Velocity in m/s). Here, Dynamic viscosity = Pa-sec = N-s/m^2^ or (N/m^2^) × sec. The constants in this calculation work out to 1.85 × 10^-6^ N-s/m. Therefore, in order to determine force, we need to determine the velocity in um/sec and multiply by 1.85 to get the force in picoNewtons (pN). We used 2.8 µm bead for all our experiments.

***Quantification of fluorescence at FA***

The fluorescence intensity difference (fold-change) between either mEmerald-Vcn or mRFP-MVcn inside the FA and outside the FA (the cytoplasm) was quantified in order to determine how much Vcn or MVcn was localized to each adhesion. We calculated these values on a per adhesion basis using ImageJ, and quantified at least 100 adhesions total (focused on adhesions at the cell periphery). The FAs stained with paxillin were thresholded as a region of interest. We then quantified the amount of either vinculin- or metavinculin-fluorescence within that same region and normalized with vinculin- or metavinculin-fluorescence in the region outside of the adhesion. The vinculin-fluorescence found within FAs relative to vinculin-fluorescence outside of the adhesions (in cytoplasm) was measured. The same quantification was performed for mRFP-MVcn fluorescence within and outside of FAs. The fluorescence intensity values for adhesions (both inside and outside) were controlled for background noise (background intensity subtraction). The fold change was calculated by determining the ratio of fluorescence intensity inside the adhesion to the fluorescence intensity outside of the adhesion. The calculation for fluorescence intensity fold-change for each adhesion was based on equal area of the adhesion (both inside and outside).
