# Supplementary material for: Vinculin and metavinculin exhibit distinct effects on focal adhesion properties, cell migration, and mechanotransduction
Source: PLoS One. 2019 Sep 4;14(9):e0221962. doi: 10.1371/journal.pone.0221962 (PMC6726196; doi:10.1371/journal.pone.0221962)

S1 Figure. mEmerald-Vcn and mRFP-MVcn cells were sorted for expression level using Flow activated cell sorting (FACS).

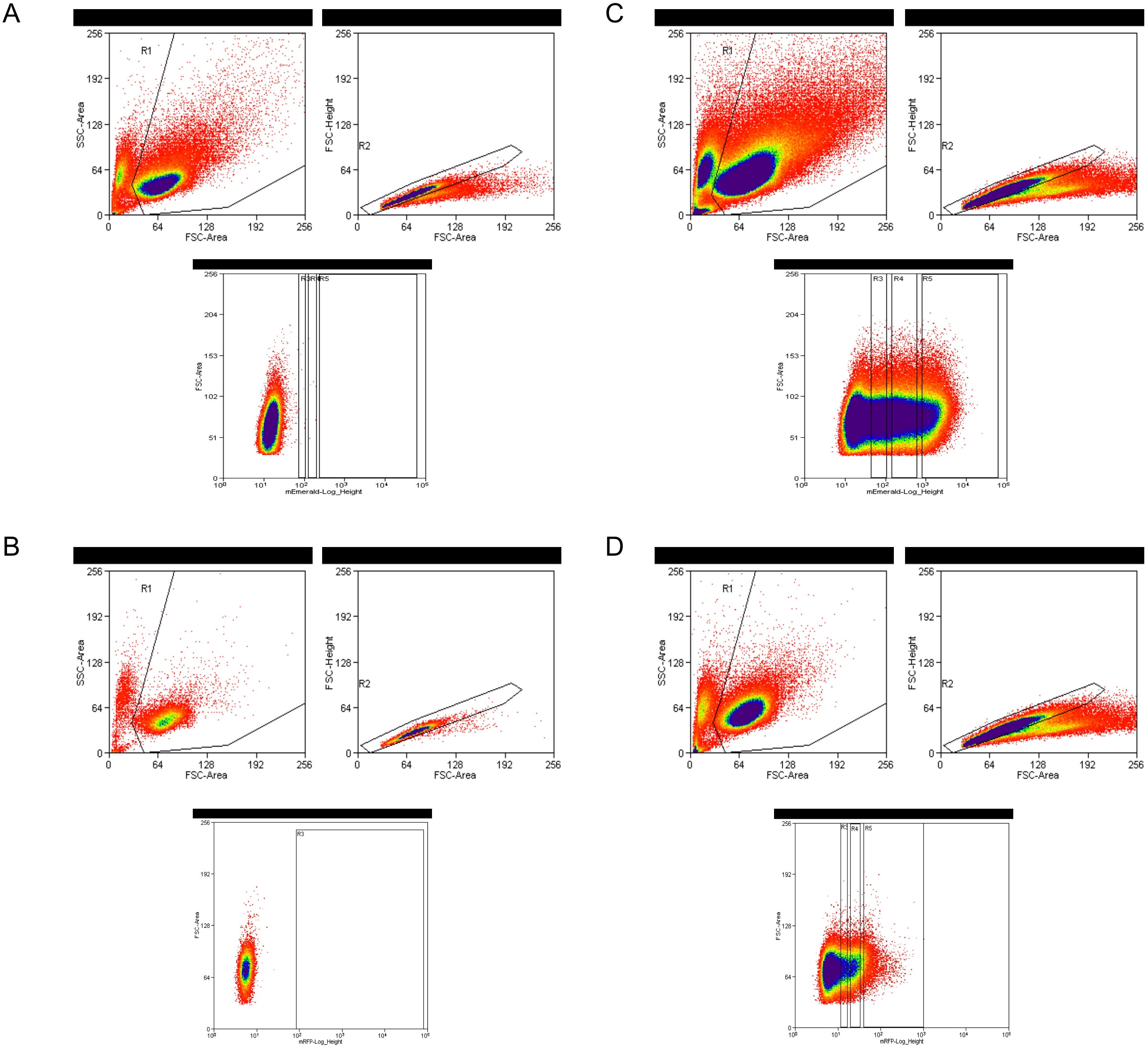

Supplement: S1 Fig — For all panels, the top left figure represents population gated for cells of interest, top right figure represents population gated for doublet discrimination, and the bottom figure represents populations sorted based on the fluorescence intensity. (A) Sort data for Vcn-null MEF as non-fluorescent control for mEmerald-tag. (B) Sort data for Vcn-null MEF as non-fluorescent control for mRFP-tag. (C) Sort data for mEmerald-Vcn cell population. Population of high-expressing mEmerald fluorescence from gate R5 used for experiments. (D) Sort data for mRFP-MVcn cell population. Population of high-expressing mRFP fluorescence from gate R5 used for experiments. (PDF) [file pone.0221962.s004.pdf]
