# Supplementary figures and images for: Vinculin and metavinculin exhibit distinct effects on focal adhesion properties, cell migration, and mechanotransduction
Source: PLoS One. 2019 Sep 4;14(9):e0221962. doi: 10.1371/journal.pone.0221962 (PMC6726196; doi:10.1371/journal.pone.0221962)

S2 Figure. Cell aspect ratio of all cell types.

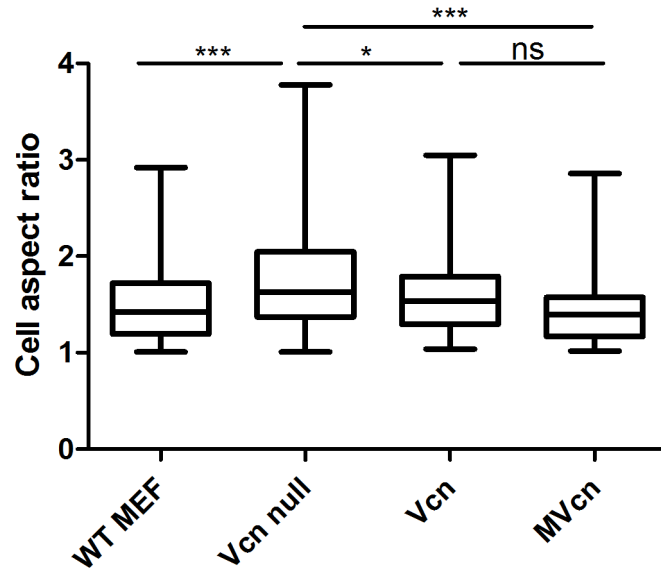

Supplement: S2 Fig — Data pooled from 3 independent experiments (n ≥ 90 cells); ***, p<0.001). (PDF) [file pone.0221962.s005.pdf]

S3 Figure. Average and representative assembly and disassembly rates at FA.

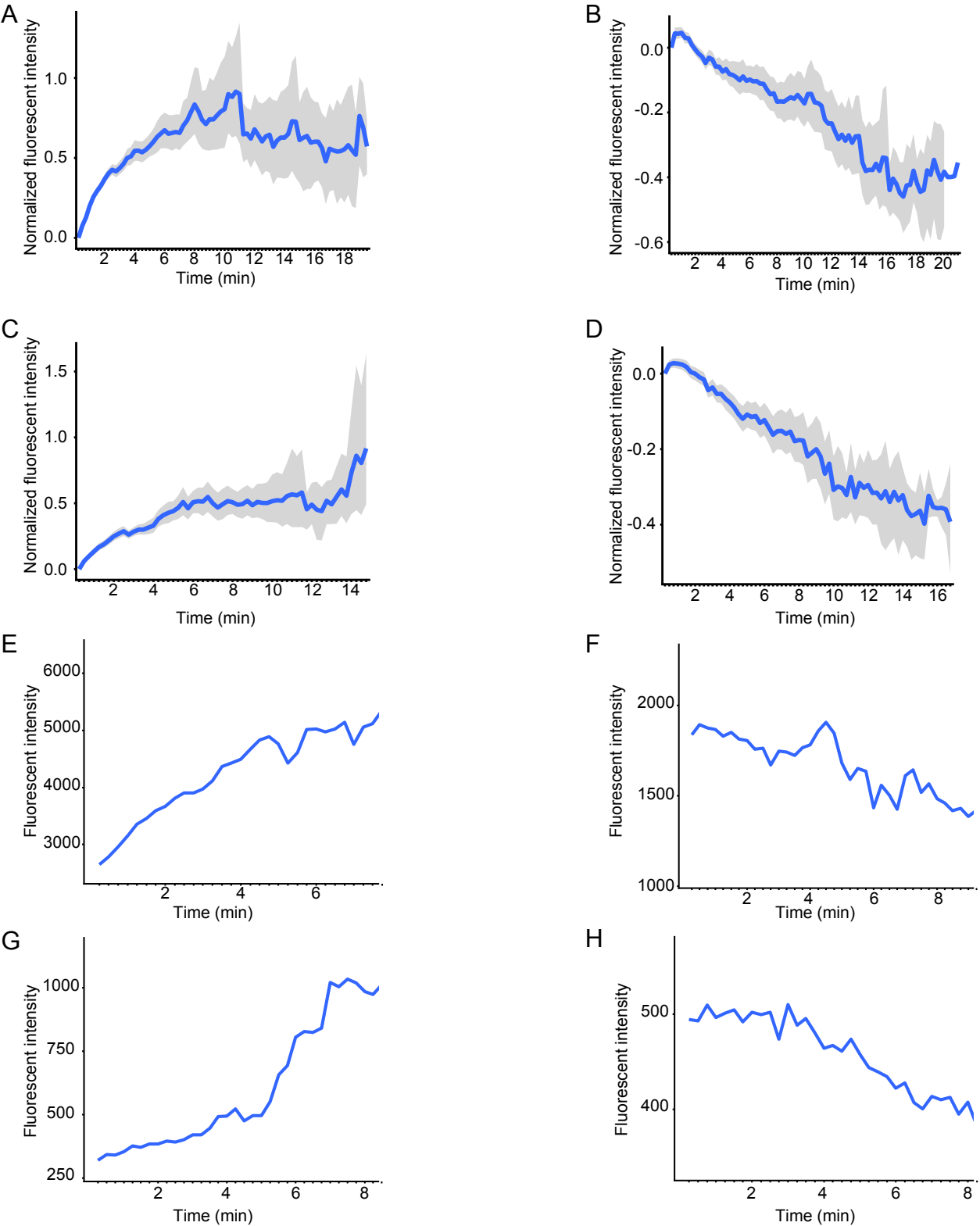

Supplement: S3 Fig — All panels show fluorescence intensity plotted with respect to time as FAs either assembled or disassembled. For (A)-(D), all shaded areas indicate S.E.M. (A) Average assembly plot of mEmerald-Vcn at FAs for all cells. (B) Average disassembly plot of mEmerald-Vcn at FAs for all cells. (C) Average assembly plot of mRFP-MVcn at FAs for all cells. (D) Average disassembly plot of mRFP-MVcn at FAs for all cells. (E) Representative assembly plot of mEmerald-Vcn at FAs for a single cell. (F) Representative disassembly plot of mEmerald-Vcn at FAs for a single cell. (G) Representative assembly plot of mRFP-MVcn at FAs for a single cell. (H) Representative disassembly plot of mRFP-MVcn at FAs for a single cell. Data pooled from 3 independent experiments (n ≥ 13 cells (or at least 500 adhesions); *p<0.05; ***, p<0.001; not significant (n.s.)). (PDF) [file pone.0221962.s006.pdf]

S5 Figure. 3DFM experimental set-up and controls.

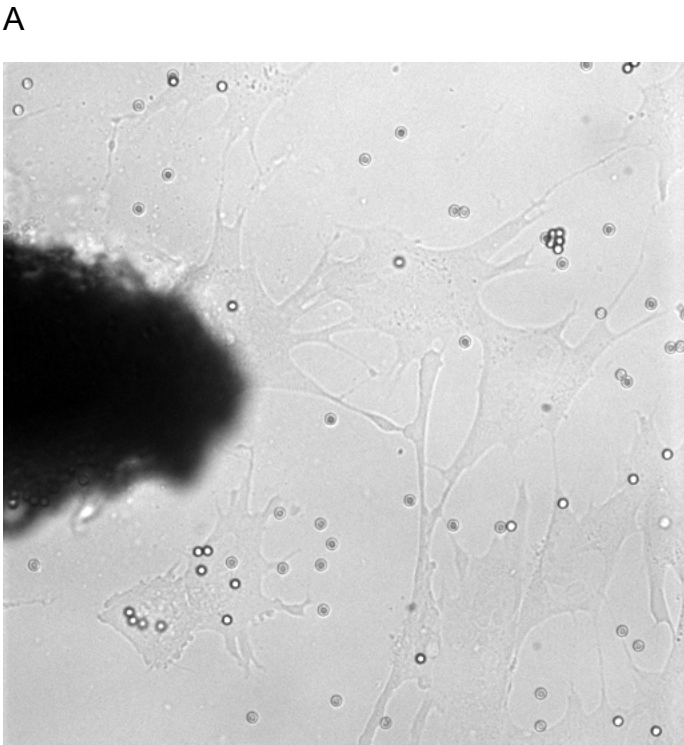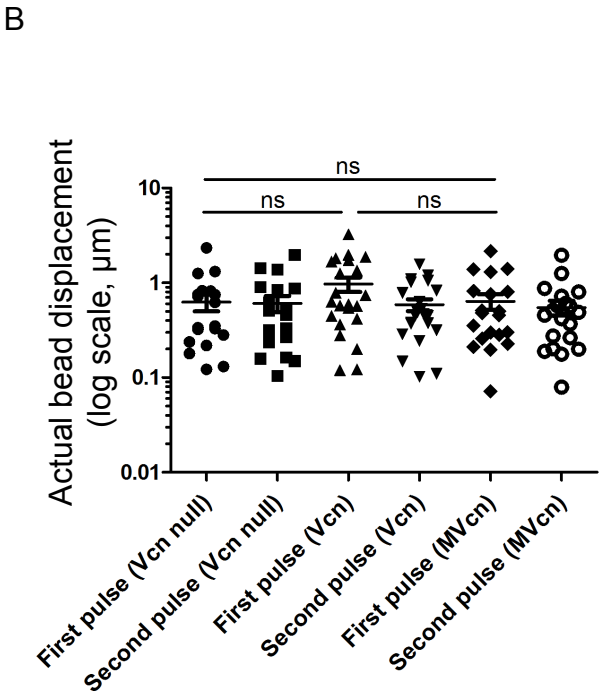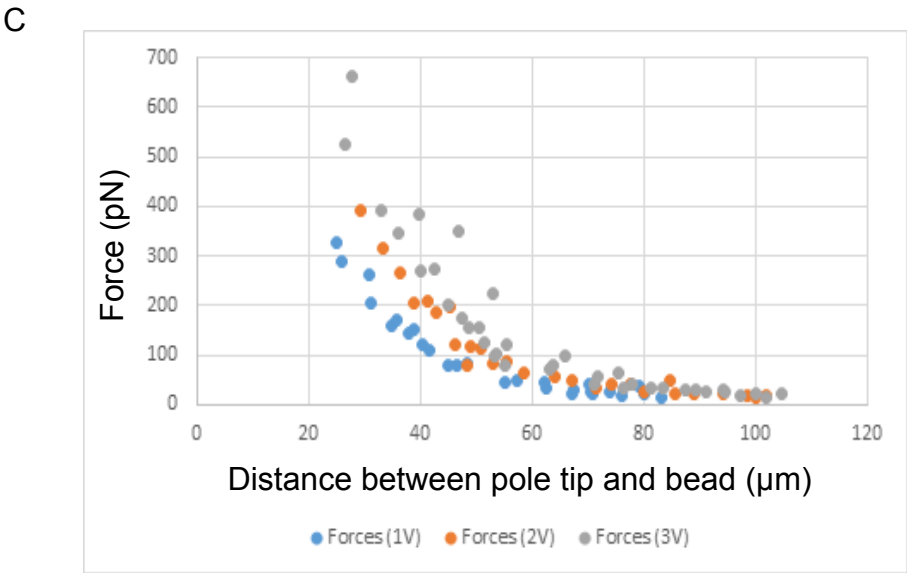

Supplement: S5 Fig — (A) Actual image of the experimental set-up. (B) Comparison of actual bead displacements between the first and second pulses for all cell types. Actual bead displacements between the first pulses of all cell types are similar. (C) Graph showing the relationship between the magnetic force experienced by the bead and the distance between the magnetic pole tip and the bead. Data pooled from 3 independent experiments (n ≥19 cells each cell type; not significant, n.s.). (PDF) [file pone.0221962.s008.pdf]

S6 Figure. Quantification for Western blot in Fig 2A.

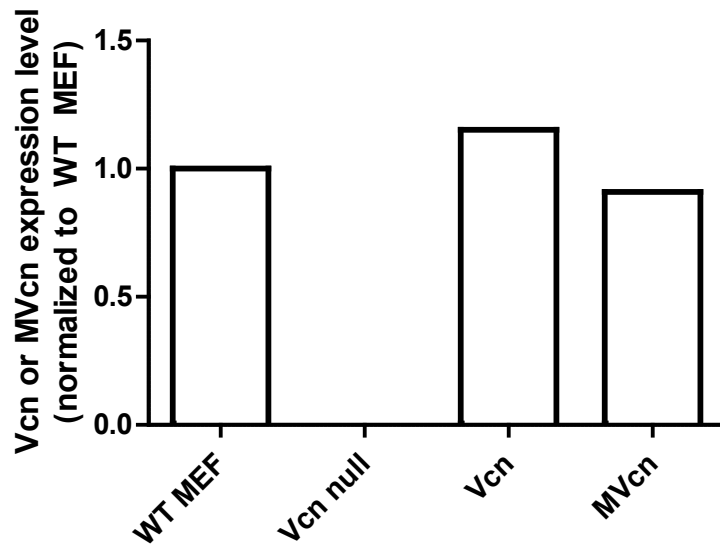

Supplement: S6 Fig — (PDF) [file pone.0221962.s009.pdf]

S7 Figure. Quantification for FA localization of mEmerald-Vcn and mRFP-MVcn.

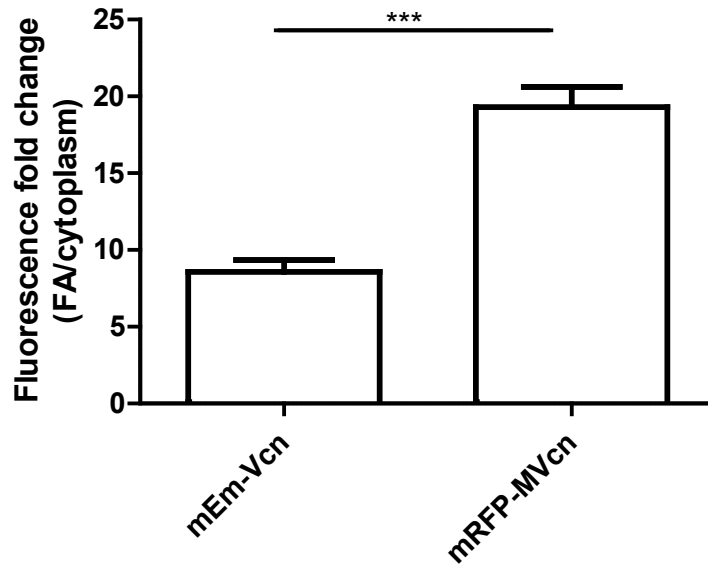

Supplement: S7 Fig — (PDF) [file pone.0221962.s010.pdf]

S8 Figure. Paired data set for each cell type in S5B Fig between first and second pulse.

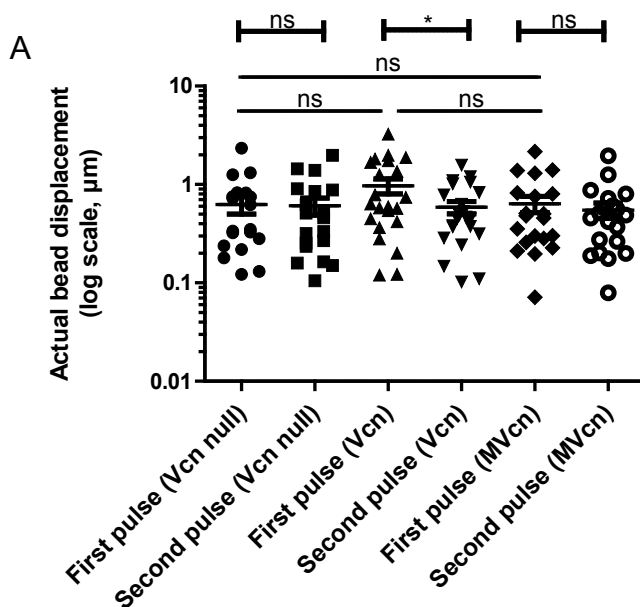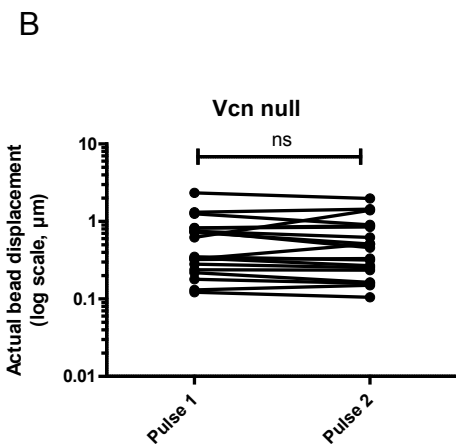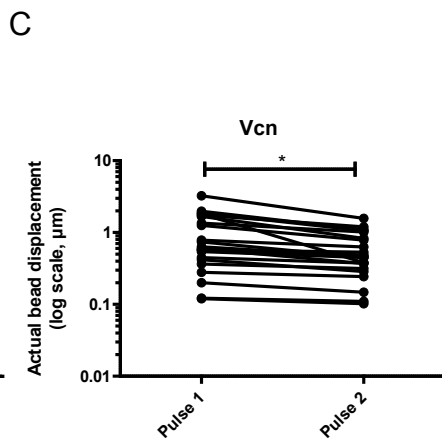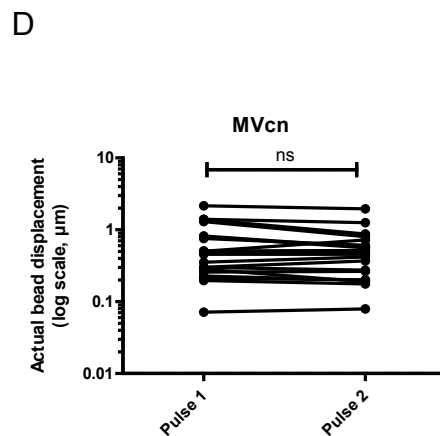

Supplement: S8 Fig — (A) Data from S5B Fig showing statistical significance between the actual bead displacements of first and second pulses for each cell type. Paired data set for actual bead displacements between first and second pulse of (B) Vcn null MEFs, (C) Vcn-expressing MEFs, and (D) MVcn-expressing MEFs are shown. Same data from (A), S5B Fig, and Fig 6 used for the analysis of (B)-(D). (PDF) [file pone.0221962.s011.pdf]

S9 Figure. Images of 3DFM set-up showing the microscope and the magnet.

A

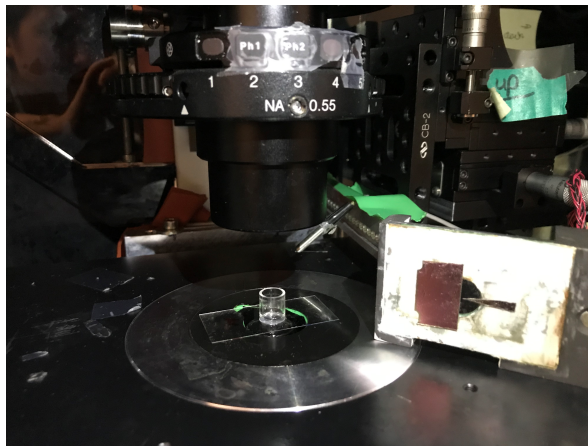

B

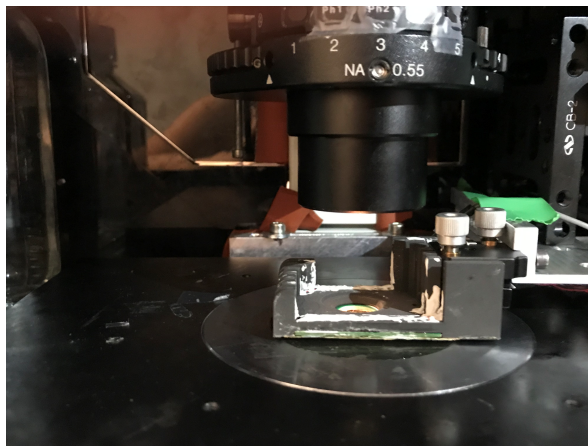

Supplement: S9 Fig — (A) Image of 3DFM microscope with cells. (B) Image of experimental set-up after the magnet has been placed. (PDF) [file pone.0221962.s012.pdf]
