# Supplementary material for: Vinculin and metavinculin exhibit distinct effects on focal adhesion properties, cell migration, and mechanotransduction
Source: PLoS One. 2019 Sep 4;14(9):e0221962. doi: 10.1371/journal.pone.0221962 (PMC6726196; doi:10.1371/journal.pone.0221962)

S4 Figure. FA assembly and disassembly rates for stably expressed mEmerald-Vcn and mEmerald-MVcn cells.

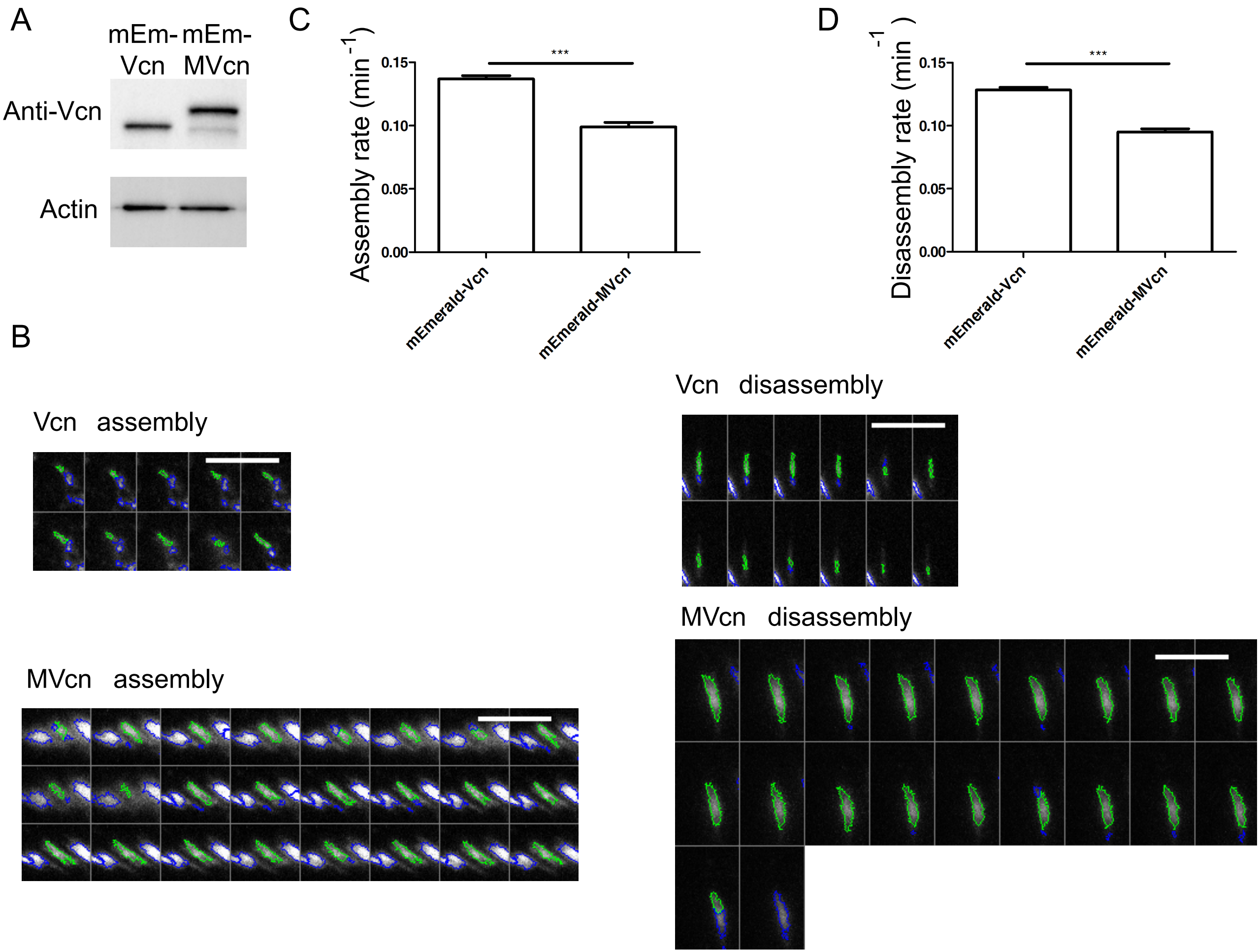

Supplement: S4 Fig — (A) Western blot shows equivalent expression level of either mEmerald-Vcn or mEmerald-MVcn in Vcn-null MEF background. (B) Representative time-lapse image sequences of Vcn-null MEFs stably expressing either mEmerald-Vcn or mEmerald-MVcn migrating on 10 μg/ml FN. Images are taken every 15 sec and show individual FA. Scale bar = 10 μm. Graph showing average rate constants of FA assembly (C) and disassembly (D) in each cell type. Data pooled from 3 independent experiments (n ≥ 13 cells (or at least 500 adhesions); ***, p<0.001). (PDF) [file pone.0221962.s007.pdf]
